# Determinants of hospitalization for a cutaneous injection-related infection among injection drug users: a cohort study

**DOI:** 10.1186/1471-2458-10-327

**Published:** 2010-06-09

**Authors:** Elisa Lloyd-Smith, Evan Wood, Ruth Zhang, Mark W Tyndall, Sam Sheps, Julio SG Montaner, Thomas Kerr

**Affiliations:** 1 British Columbia Centre for Excellence in HIV/AIDS, Providence Health Care, 608-1081 Burrard Street, Vancouver, V6Z 1Y6, Canada; 2 School of Public and Population Health, University of British Columbia, 2206 East Mall, Vancouver, V6T 1Z3, Canada; 3 Department of Medicine, University of British Columbia, 10203-2275 Laurel Street, Vancouver, V5Z 1M9

## Abstract

**Astract:**

**Background**

Cutaneous injection-related infections (CIRI) are a primary reason individuals who inject drugs (IDU) are hospitalized. The objective of this study was to investigate determinants of hospitalization for a CIRI or related infectious complication among a cohort of supervised injection facility (SIF) users.

**Methods**

From 1 January 1 2004 until 31 January 2008, using Cox proportional hazard regression, we examined determinants of hospitalization for a CIRI or related infectious complication (based on ICD 10 codes) among 1083 IDU recruited from within the SIF. Length of stay in hospital and cost estimates, based on a fully-allocated costing model, was also evaluated.

**Results**

Among hospital admissions, 49% were due to a CIRI or related infectious complication. The incidence density for hospitalization for a CIRI or related infectious complication was 6.07 per 100 person-years (95% confidence intervals [CI]: 4.96 - 7.36). In the adjusted Cox proportional hazard model, being HIV positive (adjusted hazard ratio [AHR] = 1.79 [95% CI: 1.17 - 2.76]) and being referred to the hospital by a nurse at the SIF (AHR = 5.49 [95% CI: 3.48 - 8.67]) were associated with increased hospitalization. Length of stay in hospital was significantly shorter among participants referred to the hospital by a nurse at the SIF when compared to those who were not referred (4 days [interquartile range {IQR}: 2-7] versus 12 days [IQR: 5-33]) even after adjustment for confounders (*p *= 0.001).

**Conclusions**

A strong predictor of hospitalization for a CIRI or related infectious complication was being referred to the hospital by a nurse from the SIF. This finding indicates that nurses not only facilitate hospital utilization but may provide early intervention that prevents lengthy and expensive hospital visits for a CIRI or related infectious complication.

## Background

Cutaneous injection-related infections (CIRI), which include cellulitis and abscesses, are among the primary causes of hospitalization among individuals who inject drugs (IDU) [1-4]. Hospitalizations due to CIRI carry considerable economic burden [3,5,6]. Complications of CIRI that are more likely to require hospitalization include, but are not limited to: osteomyelitis [7], bacteremia and sepsis [8,9], endocarditis [10,11], septic arthritis [7,12], ulcer [9], thrombophlebitis [13,14] and myositis [9].

A recent report by Hope et al. (2008) suggested that healthcare associated costs for CIRI among IDU in England were substantial, ranging from £15.5 million to £30.0 million per annum [5] ($27.1 to $52.4 million Canadian dollars). In 2001, Palepu et al. reported that of the IDU seen at St. Paul's Hospital, an urban hospital in Vancouver, Canada, 35% had been hospitalized and a third of these hospitalizations were due to a CIRI or related infectious complication [1]. Hospitalization was expensive with hospital utilization cost per day reported to be $610 Canadian dollars (C$610) (95% confidence interval {CI}: C$C576- C$645) [1].

North America's first supervised injection facility (SIF) opened in Vancouver's Downtown Eastside (DTES) in 2003. Within the SIF, IDU can inject pre-obtained drugs under the supervision of nurses. Individuals visiting the SIF are provided with sterile injecting equipment and emergency intervention in the event of an overdose, as well as primary medical care and addiction treatment, either on site or through referral. While several studies have pointed to the positive impact of the SIF on public disorder [15], HIV risk behaviour [16], management of overdose [17,18] and use of addiction treatment [19], its role with regard to hospitalization for CIRI remains unknown. However, recent research highlights that CIRI are a medical issue of concern among users of the SIF. Over a two year period, 6-10 per cent of IDU had a CIRI at time of interview [20]. In the present analysis, using longitudinal data, we examined predictors and cost of hospitalization for a CIRI or related infectious complication among IDU using the SIF.

## Methods

### Design and participants

The SIF in Vancouver is being evaluated through the Scientific Evaluation of Supervised Injection (SEOSI) cohort, which has been described in detail [21]. Briefly, the cohort was assembled through random recruitment of IDU from within the SIF. Random recruitment is based on inviting users of the SIF to be referred to the research study during random blocks of time. Among individuals who were recruited, a venous blood sample was drawn and an interviewer-administered questionnaire was conducted at baseline and at semi-annual follow-up visits. The informed consent agreement, obtained for all participants, included a request to link the SIF evaluation with administrative health databases. In Vancouver, hospitals are equipped with a database that tracks patient admission. The SIF is also equipped with a similar database. At the SIF, nursing care includes wound care, dressing changes, and measuring temperature. However, there are no physicians at the SIF. If microbiological investigation or medical treatment, including antibiotic prescription or intravenous antibiotic therapy is required, individuals at the SIF must be referred to the hospital or a medical clinic. In this study, a linkage of SEOSI participant data, SIF data and St. Paul's Hospital inpatient data was performed. St. Paul's Hospital is the major urban hospital serving the DTES community, one of Canada's poorest postal codes. The University of British Columbia-Providence Health Care Research Ethics Board approved the present study.

### Measurements

The start point for these analyses was enrollment into the SEOSI cohort and the endpoint was hospitalization for a CIRI or related infectious complication. The infectious complications included were based on previous literature [8,9]. The definition of the reason for hospitalization was based on International Classification of Diseases (ICD) 10 codes on patients' hospital records and included: abscess (G061, G062, L020, L021, L022, L024, J851), cellulitis (L0300, L0310, L0311, L032, L0335, L038), osteomyelitis (M4620, M4625, M4629, M8617, M8618, M8661, M8663, M8666, M8681, M8691, M8695), staphylococcal infection 161 {(A490, A499, B956) including, septicaemia (A410, A412, A419) and Methicillin-Resistant *Staphylococcus aureus *(MRSA), (U000)}, endocarditis (I330), septic arthritis (M0000, M0002, M0004, M0005, M0006, M0008, M0009), ulcer (L089, L979), thrombophlebitis (I802, I808) and myositis (M6005, M6008). A few ICD-10 codes chosen suggest but do not require infectious etiologies (e.g., thrombophlebitis, myositis, and ulcer). The number of events refers to a CIRI or related infectious complication as a primary, secondary, tertiary, quaternary, or quinary diagnoses according to relevant ICD 10 codes unless otherwise specified.

We first examined the distribution and frequency of a CIRI or related infectious complication. Then, we evaluated length of stay in hospital among study participants and examined this outcome as a continuous variable in a linear model that adjusted for the following confounding variables: age, sex, HIV serostatus and SIF nurse referral. We then considered the cost of hospitalization, associated with CIRI, which was estimated at C$712 per hospital day, based on a fully-allocated costing model for the province of British Columbia from 2001 [1]. This estimate was updated to Canadian dollars in 2005 [22]. Fully-allocated costing includes costs associated with nursing care, medications, investigations, physician visits and length of stay as well as overhead, opportunity cost of hospital resources and a 5% depreciation of capital equipment [1]. Potential healthcare savings were estimated by multiplying the cost per day value (C$712) by the difference in number of days hospitalized among individuals with CIRI who were referred by a nurse within the SIF and those who were self referred to the hospital.

We investigated baseline characteristics stratified by hospitalization or not bivariately. Using Cox proportional hazard regression, we examined factors potentially associated with hospitalization. Variables considered for our analyses included: age; sex at birth (female vs. male); current residence in DTES (yes vs. no); living in unstable housing (yes vs. no); daily cocaine injection (yes vs. no); daily heroin injection (yes vs. no); daily speedball injection (yes vs. no); and HIV serostatus (positive vs. negative). As used previously, unstable housing was defined as living in a single room occupancy (SRO) hotel, shelter, recovery or transition house, jail, on the street, or having no fixed address [23]. Variables from the semi-annual questionnaire referred to behaviour that occurred in the last six months unless otherwise specified. We also examined whether a SIF nurse referral to the hospital was associated with hospitalization and, if so, whether length of stay was different given referral versus self referral. For this task, we conducted a record linkage matching their SEOSI identifying code to each participant's record in the SIF database to determine nurse referral. Then, we linked his or her SEOSI identifying code with his or her unique personal health number to examine hospital records prior to the censor or event date.

Variable selection was based on previously published literature on a CIRI or related infectious complication and hospitalization among IDU [1,5,8,9,20]. Variables considered associated with hospitalization were analyzed in unadjusted analyses and adjusted Cox proportional hazard regression model. Time zero was defined as the date of recruitment into the SEOSI study for all participants and participants not hospitalized at St. Paul's Hospital were censored as of 31 January 2008. All behavioral variables were treated as time-updated covariates based on semi-annual follow-up data. The multivariate model was fit using a fixed model whereby we included all variables that were statistically significant at the *p *< 0.05 level in univariate analyses. All statistical analyses were performed using SAS 8.0 (Cary, NC) and all *p*-values were two-sided.

## Results

During the study period (1 January 2004 to 31 January 2008), 1083 individuals were recruited into the SEOSI cohort and 901 (83%) reported at least one follow-up visit. The median age among SEOSI participants was 38.4 years (interquartile range {IQR}: 32.7-44.3) and 314 (29%) were female. The median follow-up duration after recruitment into the cohort was 21.4 months (IQR: 13.1-24.6). During the study period, 99 (9%) participants were admitted to St. Paul's Hospital, yielding an incidence density of hospitalization for a CIRI or related infectious complication of 6.07 per 100 person-years (95% CI: 4.96 - 7.36). Among all hospital admissions among SEOSI participants, 49% (216 of 442) were related to a CIRI or related infectious complication. A total of 216 hospitalization events occurred among 99 individuals for a CIRI or related infectious complication. Among the 99 hospitalized SEOSI participants, 47 received CIRI care at the SIF during the study period. A total of 145 SEOSI participants were referred by a nurse at the SIF while 63 participants were referred and visited the ED. In addition, 115 SEOSI participants were referred but subsequently not hospitalized. Importantly, 60% (28/47) of those who received CIRI care at the SIF were referred to the hospital by a nurse at the SIF and subsequently sought treatment at the hospital. Individuals with an ED visit who were referred by a nurse were more likely to be hospitalized within three days (*p *= 0.011). Further, those who were referred to the hospital by a nurse at the SIF were significantly more likely to have been seen by a nurse at the SIF for a CIRI (8 nurse visits for a CIRI [IQR: 3-16]) compared to those that were not referred to a hospital (1 nurse visit for a CIRI [IQR: 0-4]) (*p *< 0.001). Virtually all patients are admitted to the hospital from the Emergency Department (ED).

Tables 1, 2 and 3 display the frequency of a CIRI or related infectious complication. Cellulitis was the most common reason for hospitalization. Fifteen persons had missing data on HIV serostatus and were excluded from analyses. Therefore, all results on HIV were based on a sample size of 1068. Participants who had been referred to the hospital by an SIF nurse had a significantly shorter length of stay in hospital as compared to those who were not referred (4 days [IQR: 2-7], 12 days [IQR: 5-33]). The eight day reduction in the length of hospital stay remained significant (*p *= 0.001) after adjustment for confounding variables. Considering the total potential healthcare saving based on a fully allocated hospital cost per day calculation, each referral from the SIF would have resulted in a saving of C$5,696 (IQR: C$2, 136 - C$18, 512).

**Table 1:** Description of hospitalizations for a cutaneous injection-related infection or related infectious complication among Scientific Evaluation of Supervised Injection participants.

**Classification**^**†**^	First event	Total events
	n (%)	n (%)
	n = 99	n = 216
**Cellulitis**	33 (33)	59 (27)
**Abscess**	14 (14)	26 (12)
**Osteomyelitis**	10 (10)	39 (18)
**Staphylococcal infection**	22 (22)	42 (17)
**Endocarditis**	9 (9)	24 (12)
**Septic arthritis**	7 (7)	12 (6)
**Ulcer**	1 (1)	6 (3)
**Thrombophlebitis**	1 (1)	4 (2)
**Myositis**	2 (2)	4 (2)

† Classification is based on International Classification of Diseases 10 codes

**Table 2:** Distribution of hospitalizations for a cutaneous injection-related infection or related infectious complication*.

	Primary diagnosis	+1	+2	+3	+4	+5	+6	+7	+8	+9	+1&2	+1&4
**1cellulitis**	45	-	3	0	5	0	0	2	1	0	-	-
**2abscess**	9	1	-	0	5	0	0	0	0	0	-	3
**3osteomyelitis**	14	0	5	-	5	0	0	0	1	2	0	1
**4staph.infection**	18	3	0	1	-	4	0	0	0	0	1	-
**5endocarditis**	12	0	1	0	5	-	0	0	0	0	0	1
**6septic arthritis**	8	0	2	0	1	0	-	0	0	0	0	1
**7ulcer**	3	0	0	0	1	0	0	-	0	0	0	0
**8thrombophlebitis**	3	1	0	0	0	0	0	0	-	0	0	0
**9myositis**	1	0	0	0	3	0	0	0	0	-	0	0

	+1&7	+2&4	+3&4	+3&7		+4&7		+5&4	+9&4		+ more than two diseases	

**1cellulitis**	-	0	0	1		2		0	0		0	
**2abscess**	0	-	2	0		0		1	1		4	
**3osteomyelitis**	1	3	-	-		2		0	0		5	
**4staph.infection**	0	-	-	0		-		-	-		15	
**5endocarditis**	0	0	0	0		0		-	0		5	
**6septic arthritis**	0	0	0	0		0		0	0		0	
**7ulcer**	-	0	0	-		-		0	0		2	
**8thrombophlebitis**	0	0	0	0		0		0	0		0	
**9myositis**	0	0	0	0		0		0	-		0	

*According to ICD 10 codes. ICD 10 codes categorized into 9 diseases and listed from 1 to 9. First column refers to when disease listed as primary diagnosis. Other columns refer to multiple diseases reported, irrespective of whether event was primary, secondary, tertiary, quaternary, or quinary.

**Table 3:** Frequency of hospitalizations for a cutaneous injection-related infection or related infectious complication*.

	Once	Twice	Three times	Four times	Five plus times	Total
**1cellulitis**	19	7	3	0	2	59
**2abscess**	10	5	2	0	0	26
**3osteomyelitis**	5	2	2	0	3	39
**4staph.infection**	10	2	0	3	2	42
**5endocarditis**	11	0	1	0	1	24
**6septic arthritis**	6	3	0	0	0	12
**7ulcer**	1	0	1	0	1	6
**8thrombophlebitis**	1	0	1	0	0	4
**9myositis**	3	0	1	0	0	4

*According to ICD 10 codes. Diseases listed from 1 to 9. ICD 10 codes categorized into 9 diseases and listed from 1 to 9. Data relates to number of times an individual developed a disease, irrespective of whether event was coded as primary, secondary, tertiary, quaternary, or quinary.

The factors associated with an increased risk of hospitalization after recruitment into the SEOSI cohort are shown in Table 4. In the multivariate model being HIV positive (adjusted hazard ratio [AHR] = 1.79 [95% CI: 1.16 - 2.75]) and being referred to the hospital by a nurse at the SIF (AHR = 5.38 [95% CI: 3.39 - 8.55]) were positively and independently associated with an increased likelihood of hospitalization.

**Table 4:** Univariate and multivariate Cox proportional hazard analyses of hospitalizations for a cutaneous injection-related infection or related infectious complication among among Scientific Evaluation of Supervised Injection participants.

	Unadjusted			Adjusted		
	Hazard Ratio (HR)			Hazard Ratio (AHR)		
Variable	HR	(95% CI)	*p*-value	AHR	(95% CI)	*p*-value
**Age**						
(per year older)	0.98	(0.96 - 1.01)	0.146			
**Sex**						
(Female vs Male)	1.59	(1.07 - 2.39)	0.024	1.36	(0.90 - 2.05)	0.139
Unstable housing*						
(Yes vs No)	1.65	(1.08 - 2.53)	0.021	1.26	(0.79 - 2.02)	0.328
Cocaine injection*						
(Daily vs Not daily)	1.75	(1.17 - 2.62)	0.006	1.46	(0.94 - 2.25)	0.090
Speedball injection*						
(Daily vs Not daily)	1.90	(1.15 - 3.14)	0.012	1.19	(0.69 - 2.07)	0.528
**HIV serostatus**						
(Positive vs Negative)	2.12	(1.39 - 3.24)	<0.001	1.79	(1.16 - 2.75)	0.008
**Hospital referral†**						
(Yes vs No)	2.41	(1.55 - 3.77)	<0.001	5.38	(3.39 - 8.55)	<0.001

*Behaviour refers to activities in the last 6 months. †Indicates data derived from SIF database and by a study nurse. CI = confidence interval.

## Discussion

In the present study, being referred by a nurse at a SIF was independently associated with an elevated rate of hospitalization. Importantly, participants who had been referred to the hospital by a nurse at the SIF had a significantly shorter length of stay in hospital despite adjustment for HIV infection and other potential confounders. This finding indicates that nurses facilitate hospital utilization as well as providing early intervention that prevents lengthy and expensive hospital visits for a CIRI or related infectious complication. According to the cost savings calculation based on the length of stay reduction, SIF nurse referral resulted in a minimum saving of C$2,136 per admission. However, it is important to note that variability in treatment costs exists in the CIRI and related infectious complications included in this investigation.

Using the same cohort, the risk factors for the outcome of developing a CIRI have been reported to include being female, living in unstable housing, borrowing syringes, requiring help injecting and injecting cocaine daily [20]. Some factors differ from risk factors in other cities. For example, research from San Francisco reports an associated between developing a CIRI and frequent black tar heroin injection [2] whereas in Vancouver, frequent cocaine injection has been associated with developing a CIRI [20]. The present study evaluated the predictors of the outcome of hospitalization for a CIRI or related infectious complication. The predictors of hospitalization for a CIRI or related infectious complication included being HIV positive and referred to the hospital by a nurse at the SIF. Future research investigating outcomes, including mortality from a CIRI or related infectious complication among those referred compared to those not referred to the hospital by a nurse at the supervised injection facility would support our understanding of the potential impact of nurse referral. Interestingly, being HIV positive was not a risk factor for the development of CIRI but was a predictor of hospitalization for a CIRI or related infectious complication.

As noted above, an increased risk of hospitalization for a CIRI or related infectious complication was observed among HIV positive participants. This finding is consistent with previous research on hospitalization among IDU in this setting [1]. Individuals with HIV may be more likely to be treated as an inpatient as opposed to an outpatient (i.e., HIV positive patients with abscesses may be more likely to get admitted to the hospital than HIV negative patients with abscesses). A potentially elevated susceptibility to bacterial infections [8,24] as well as known high-risk drug injection practices in this subpopulation [25,26] may related to why HIV positive participants in this study had elevated rates of hospitalization. Further elucidation of this finding with a larger sample of HIV positive IDU is necessary to examine this important research question.

After controlling for factors that are known to be associated with hospitalization [1,27], referral from an SIF nurse remained a strong predictor of hospitalization. Findings from this study confirm existing qualitative research indicating that nurses at the SIF provide CIRI-related care and play a key role in enhancing access to medical care among participants who have infections or injuries of greater severity [28]. Hospital care for more severe forms of CIRI is essential since local hospitals provide treatment to CIRI not available at the SIF (e.g., incision and drainage of an abscess, intravenous antibiotic therapy as well as diagnostic tools and therapy appropriate for complications). Further, given that the average length of stay in hospital for those referred to the hospital by a nurse at the SIF was significantly shorter, it may be that nurses at the SIF are helping reduce the incidence of late presentation of complications, such as osteomyelitis, that are often lengthy and are particularly costly to the healthcare system [7]. Although our study design does not allow us to infer causation, it is noteworthy that this association persisted in multivariate analyses. This may reflect prompt referral as well as the preventative effect of the SIF on serious infections [16].

There are several examples of efficient and cost effective community-based treatment services that may inform changes in the provision of care that is required in order to reduce the incidence of hospitalization of a CIRI or related infectious complication in our setting. In its first year of operation, the Integrated Soft Tissue Infection Services (ISIS) Clinic in San Francisco, California, resulted in a 47% decrease in surgical service admissions and an estimated savings of over $8.0 (C$8.4) million for costs related to CIRI [29]. In addition, a wound management clinic operated in conjunction with a syringe exchange program in Oakland, California [30]. It was found that the average cost per individual treated at this wound management clinic was $5.0 (C$5.2), substantially lower than equivalent hospital costs of $185.0 (C$193.4) and $360.0 (C$376.3) [30]. Given the success other cities have had in implementing additional medical treatment for CIRI in the community, we recommend initiating provision of on-site incision and drainage for abscesses and administration of antibiotic therapy at the SIF or in a nearby community setting. Further, to prevent development of CIRI, it may be of value to screen and treat for MRSA at the SIF or at the SEOSI research site. In our setting, expanding the capacity to provide primary care in an integrated manner is warranted.

There are limitations of the present study to consider when interpreting this data. Firstly, St. Paul's Hospital was the only facility linkage that was conducted. However, this is the primary hospital serving the SIF catchment area and is accessed extensively by the IDU population in the DTES [1,27,31]. Secondly, our study relies on self report to obtain drug use and other behavioural variables. However, self report among IDU is considered valid [32] and hospital utilization and nurse referral were accessed directly from databases within the hospital and at the SIF, respectively. Thirdly, a limitation of this analysis is that a low sample size of HIV positive participants precluded our ability to conduct further analyses on this association. Additional research is required with a larger sample of HIV positive individuals to better elucidate this finding. Fourthly, additional research is required to better understand why a shorter length of stay is seen among SEOSI participants referred by a nurse at the SIF to the hospital. It is possible that nurses may be referring to the hospital SEOSI participants that require shorter lengths of stay (e.g., abscess versus osteomyelitis). However, in our study, individuals with an ED visit who were referred by a nurse were significantly more likely to be hospitalized within three days. Similarly, Binswanger et al. found ED use for CIRI to be associated with hospitalization and also death [33].

## Conclusion

In summary, we found high levels of hospitalization for a CIRI or related infectious complication among local IDU. Being HIV positive and being referred to the hospital by a nurse at the SIF, as opposed to self referral to the hospital, were both independently and positively associated with an increased likelihood of hospitalization. Participants who had been referred to the hospital by a nurse at the SIF had a significantly shorter length of stay in hospital as compared to those who were not. These findings indicate that nurses at the SIF play a critical role in terms of referring individuals who require hospitalization for a CIRI or related infectious complication to the hospital, which may result in shorter and less expensive hospital visits. Expanded management of CIRI in the community may reduce the need for referral to the hospital, further reducing the cost of caring for this common clinical problem among IDU.

## Competing interests

The authors declare that they have no competing interests.

## Authors' contributions

ELS conceived and designed the study and drafted the manuscript. RZ and ELS performed the statistical analyses. ELS, TK, EW, MT contributed to the design and coordination of the study. All authors provided assistance with interpretation of the results to the drafts of the manuscript and read and approved the final manuscript.

## Pre-publication history

The pre-publication history for this paper can be accessed here:

http://www.biomedcentral.com/1471-2458/10/327/prepub

## References

[B1] Palepu A Tyndall MW Leon H Muller J O'Shaughnessy MV Schechter MT Hospital utilization and costs in a cohort of injection drug users CMAJ 2001 165 415 420 PMC81365 11531049

[B2] Murphy E DeVita D Liu H Vittinghoff E Leung P Ciccarone DH Risk factors for skin and soft-tissue abscesses among injection drug users: a case-control study Clin Infect Dis 2001 33 35 40 10.1086/320879 11389492

[B3] Ciccarone D Bamberger JD Kral AH Edlin BR Hobart CJ Moon A Soft tissue infections among injection drug users - San Francisco, California, 1996-2000 MMWR Morb Mortal Wkly Rep 2001 50 381 384 11465906

[B4] Takahashi TA Baernstein A Binswanger I Bradley K Merrill JO Predictors of hospitalization for injection drug users seeking care for soft tissue infections J Gen Intern Med 2007 22 382 388 10.1007/s11606-006-0079-y PMC1824763 17356973

[B5] Hope V Kimber J Vickerman P Hickman M Ncube F Frequency, factors and costs associated with injection site infections: findings from a national multi-site survey of injecting drug users in England BMC Infect Dis 2008 8 10.1186/1471-2334-8-120 PMC2600824 18801177

[B6] DiNubile MJ Lipsky BA Complicated infections of the skin and skin structures: when the infection is more than skin deep J Antimicrob Chemother 2004 53 ii37 ii50 10.1093/jac/dkh202 15150182

[B7] Kak V Chandrasekar PH Bone and joint infections in injection drug users Infect Dis Clin North Am 2002 16 681 695 10.1016/s0891-5520(02)00016-8 12371122

[B8] Gordon RJ Lowy FD Bacterial infections in drug users N Engl J Med 2005 353 1945 1954 10.1056/NEJMra042823 16267325

[B9] del Giudice P Cutaneous complications of intravenous drug abuse Br J Dermatol 2004 150 1 10 10.1111/j.1365-2133.2004.05607.x 14746612

[B10] DiNubile MJ Short-course antibiotic therapy for right-sided endocarditis caused by *Staphylococcus aureus *in injection drug users Ann Intern Med 1994 121 873 876 10.7326/0003-4819-121-11-199412010-00009 7978701

[B11] Ngaage DL Cowen ME Right ventricular needle embolus in an injecting drug user: the need for early removal Emerg Med J 2001 18 500 501 10.1136/emj.18.6.500 PMC1725731 11696516

[B12] Ebright JR Pieper B Skin and soft tissue infections in injection drug users Infect Dis Clin North Am 2002 16 697 712 10.1016/s0891-5520(02)00017-x 12371123

[B13] Schulz S Beckenbach C Philipp M Hengstmann J Color coded duplex sonography of inguinal vessels in i.v. drug addicts Vasa 2002 31 7 13 10.1024/0301-1526.31.1.7 11951704

[B14] Roszler MH McCarroll KA Donovan KR Rashid T Kling GA The groin hit: complications of intravenous drug abuse Radiographics 1989 9 487 508 10.1148/radiographics.9.3.2727357 2727357

[B15] Wood E Kerr T Small W Palepu A Tyndall MW Changes in public order after the opening of a medically supervised safer injecting facility for illicit injection drug users CMAJ 2004 169 759 763 10.1503/cmaj.1040774 PMC517857 15451834

[B16] Kerr T Tyndall M Li K Montaner J Wood E Safer injection facility use and syringe sharing in injection drug users Lancet 2005 366 316 318 10.1016/S0140-6736(05)66475-6 16039335

[B17] Milloy MJS Kerr T Tyndall M Montaner J Wood E Estimated drug overdose deaths averted by North America's first medically-supervised safer injection facility PLoS ONE 2008 3 e3351 10.1371/journal.pone.0003351 PMC2556397 18839040

[B18] Milloy MJS Kerr T Mathias R Zhang R Montaner J Tyndall M Wood E Non-fatal overdose among a cohort of active injection drug users recruited from a supervised injection facility Am J Drug Alcohol Abuse 2008 34 499 509 10.1080/00952990802122457 18584579

[B19] Wood E Tyndall MW Zhang R Stoltz JA Montaner J Kerr T Attendance at supervised injection facilities and use of detoxification services N Engl J Med 2006 354 2512 2514 10.1056/NEJMc052939 16760459

[B20] Lloyd-Smith E Wood E Zhang R Tyndall M Montaner J Kerr T Risk factors for developing a cutaneous injection-related infection among injection drug users: a cohort study BMC Public Health 2008 8 405 10.1186/1471-2458-8-405 PMC2621202 19068133

[B21] Wood E Kerr T Lloyd-Smith E Buchner C Marsh D Montaner J Tyndall MW Methodology for evaluating Insite: Canada's first medically supervised safer injection facility for injection drug users Harm Reduct J 2004 1 10.1186/1477-7517-1-9 PMC535533 15535885

[B22] Organisation for Economic Co-operation and Development (OECD) Health data 2007 2007

[B23] Corneil TA Kuyper LM Shoveller J Hogg RS Li K Spittal P Schechter M Wood E Unstable housing, associated risk behaviour, and increased risk for HIV infection among injection drug users Health Place 2006 12 79 85 10.1016/j.healthplace.2004.10.004 16243682

[B24] Brettle RP Bacterial infections in HIV: the extent and nature of the problem Int J STD AIDS 1997 5 5 15 10.1258/0956462971918698 9043975

[B25] Stein MD Sobota M Injection drug use: hospital care and charges Drug Alcohol Depend 2001 64 117 120 10.1016/s0376-8716(00)00235-0 11470348

[B26] Spijkerman IJ van Ameijden EJ Mientjes GH Coutinho RA van den Hoek A Human immunodeficiency virus infection and other risk factors for skin abscesses and endocarditis among injection drug users J Clin Epidemiol 1996 49 1149 1154 10.1016/0895-4356(96)00180-1 8826995

[B27] Palepu A Strathdee SA Hogg RS Anis AH Rae S Cornelisse PG Patrick DM O'Shaughnessy MV Schechter MT The social determinants of emergency department and hospital use by injection drug users in Canada J Urban Health 1999 76 409 418 10.1007/BF02351499 PMC3456690 10609591

[B28] Small W Wood E Lloyd-Smith E Tyndall M Kerr T Accessing care for injection-related infections through a medically supervised injecting facility: a qualitative study Drug Alcohol Depend 2008 98 159 162 10.1016/j.drugalcdep.2008.05.014 18650034

[B29] Harris HW Young DM Care of injection drug users with soft tissue infections in San Francisco, California Arch Surg 2002 137 1217 1222 10.1001/archsurg.137.11.1217 12413304

[B30] Grau LE Arevalo S Catchpool C Heimer R Expanding harm reduction services through a wound and abscess clinic Am J Public Health 2002 92 1915 1917 10.2105/ajph.92.12.1915 PMC1447352 12453808

[B31] Kerr T Wood E Grafstein E Ishida T Shannon K Lai C Montaner JM Tyndall MW High rates of primary care and emergency department use among injection drug users in Vancouver J Public Health 2005 27 62 66 10.1093/pubmed/fdh189 15564279

[B32] Darke S Self-report among injecting drug users: a review Drug Alcohol Depend 1998 51 253 263 10.1016/s0376-8716(98)00028-3 9787998

[B33] Binswanger IA Takahashi TA Bradley K Dellit TH Benton KL Merrill JO Drug users seeking emergency care for soft tissue infection at high risk for subsequent hospitalization and death J Stud Alcohol Drugs 2008 69 924 932 10.15288/jsad.2008.69.924 PMC2583377 18925351

